# Phillygenin suppresses hepatocellular carcinoma progression by modulating the TNF signaling pathway and TCA cycle metabolism

**DOI:** 10.3389/fphar.2026.1854265

**Published:** 2026-06-12

**Authors:** Jiao Luo, Chang Liu, Yi Pang, Lianhong Pan

**Affiliations:** 1 Chongqing Key Laboratory of Development and Utilization of Genuine Medicinal Materials in Three Gorges Reservoir Area, Chongqing Engineering Research Center of Antitumor Natural Drugs, Natural Drug Intervention in Aging Key Laboratory of Chongqing Education Commission of China, Chongqing Three Gorges Medical College, Chongqing, China; 2 Chongqing University Three Gorges Hospital, Chongqing, China

**Keywords:** hepatocellular carcinoma, oxoglutarate dehydrogenase, phillygenin, TNF signaling pathway, tricarboxylic acid cycle

## Abstract

**Background:**

Phillygenin (PLE) is a bioactive constituent of *Forsythiae fructus* that exerts hepatoprotective, anti-inflammatory, and antitumor effects. However, its role in hepatocellular carcinoma (HCC) and the mechanisms underlying its activity remain insufficiently characterized.

**Methods:**

The effect of PLE on HCCLM3 and Hep3B tumor cells was assessed using immunofluorescence (IF) staining, TUNEL assay, qRT-PCR, and Western blotting experimental methodology. RNA-sequencing (RNA-seq) was used to detect changes in gene expression and to explore the gene targets and related pathways. Liquid chromatography–mass spectrometry (LC–MS) was used to identify the effects of PLE on primary metabolite targets and metabolism-related pathways. Tumor xenograft models were used for *in vivo* verification.

**Results:**

PLE inhibited HCC cell proliferation through the inhibition of cell cycle progression and induction of cell apoptosis *in vitro,* indicating a dual antiproliferative and pro-apoptotic effect. These responses were associated with the modulation of the TNF signaling pathway. Moreover, PLE induced significant alterations in the central carbon metabolism, including the regulation of citrate synthesis and upregulation of oxoglutarate dehydrogenase (OGDH) expression in HCC cells. Finally, PLE treatment significantly reduced tumor growth *in vivo*, thereby confirming its therapeutic potential.

**Conclusion:**

PLE exerts antitumor effects in HCC through the coordinated regulation of tricarboxylic acid (TCA) cycle metabolism while simultaneously inhibiting the TNF signaling pathway, highlighting its potential as a candidate antitumor drug for the treatment of HCC.

## Introduction

Hepatocellular carcinoma (HCC) is a highly prevalent malignancy with substantial morbidity and mortality worldwide, significantly compromising patient survival and quality of life (Schlosser et al., 2022). The complexity of HCC pathogenesis necessitates a multidisciplinary approach to its treatment by combining hepatology, surgery, radiology, and medical and radiation oncology (Sidali et al., 2022). Despite advances in diagnostic and strategies, clinical outcomes remain suboptimal, and the prognosis of patients with HCC continues to be poor (Couri and Pillai, 2019). Although chemotherapy and targeted therapies, including cisplatin and sorafenib, are widely used, their efficacy is often limited by the development of drug resistance (Feng et al., 2021; Pan et al., 2022). Therefore, the identification of new therapeutic targets and the development of more effective treatment strategies remain urgently needed.

Metabolic reprogramming is a hallmark of tumor development and progression, enabling tumor cells to sustain rapid proliferation and increased energy demands (Nam et al., 2024; Huang et al., 2025). The tricarboxylic acid (TCA) cycle represents a central metabolic hub that regulates ATP production and provides key biosynthetic precursors required for tumor growth (Sarkar et al., 2025). Dysregulation of metabolic pathways, including alterations in TCA cycle enzyme activity, contributes to tumor progression and metabolic adaptation (Liu et al., 2025; Zhang et al., 2025). However, although enzymes such as isocitrate dehydrogenase (IDH) or succinate dehydrogenase (SDH) have been extensively investigated (Todisco et al., 2019), the role of oxoglutarate dehydrogenase (OGDH) in HCC remains poorly defined. Targeting TCA cycle metabolism, particularly OGDH, may, therefore, represent a promising therapeutic strategy.

*Forsythiae fructus,* a traditional medicinal herb derived from the genus Forsythia of the Meliaceae family, is widely recognized for its anti-inflammatory and hepatoprotective properties (Hu et al., 2020). Phillygenin (PLE), the main bioactive component of *F. fructus*, possesses multiple pharmacological activities, including hepatoprotective (Song et al., 2018), anti-inflammatory, anti-oxidant (Du et al., 2019), and anti-tumor properties (Li et al., 2020; Ding et al., 2021). PLE attenuates inflammatory responses through the modulation of pathways such as NF-κB and NLRP3 (Zhou et al., 2021), as well as by promoting autophagy (Zhou et al., 2022). Additionally, PLE inhibits IL-1β-induced extracellular matrix (ECM) degradation and decreases pro-inflammatory cytokine production in various cellular models (Zhang et al., 2023). However, its potential tumor-inhibitory effect in HCC and the underlying mechanisms remain insufficiently characterized. In particular, it has not yet been established whether PLE modulates HCC progression through metabolic reprogramming.

Therefore, we aimed to determine the effect of PLE on HCC using *in vitro* and *in vivo* models. PLE exerted antitumor activity by inhibiting cell proliferation and migration while promoting apoptosis. Mechanistically, these effects were associated with the modulation of the TNF signaling pathway. Furthermore, PLE altered TCA cycle metabolism by regulating key metabolic intermediates and upregulating OGDH expression, suggesting a role in metabolic reprogramming. Collectively, these findings suggested that PLE suppresses HCC progression through the coordinated regulation of TNF signaling and TCA cycle metabolism, supporting its potential as a candidate antitumor drug, although further validation in clinical settings is required.

## Materials and methods

### Cell culture

Human HCC cell lines HCCLM3 and Hep3B were acquired from the Liver Cancer Institute, Fudan University (Shanghai, China). Cells were routinely cultured as previously described (Pan et al., 2021).

### Drug treatment

PLE (relative molecular mass: 372.41, Selleck, USA) was dissolved in DMSO to prepare a 50 mM stock solution. This stock was serially diluted with culture medium to obtain the working concentrations of 10, 20, and 40 μM used for the treatment of HCC cells.

### Cell viability assays

HCC cells were seeded onto a 96-well plate at a concentration of 1 × 10^4^ cells per well and cultured under low-serum conditions (2% FBS) for 12 h. Next, PLE (10, 20, or 40 μM) was added, and cells were incubated for 48 h. Subsequently, 10 μL CCK-8 solution (Dojindo, Molecular Technology, Japan) was added with 10 μL per well. The cells were incubated for 2 h, and absorbance was read at 450 nm using a microplate reader (Bio-Tek, USA).

### Clone formation assay

HCCLM3 and Hep3B cells (500 per well) were seeded onto a 6-well plate and incubated for 7 days. Then, the medium was replaced with fresh medium containing PLE (10, 20, or 40 μM) and incubated for an additional week. Next, the cells were fixed with 4% paraformaldehyde (PFA; Leagene, Beijing, China) at 37 °C for 1 h, and the colonies were stained with 0.05% crystal violet (Beyotime, China). Finally, colonies were washed, imaged, and counted.

### Western blotting analysis

Cells were treated with PLE (10, 20, or 40 μM) for 48 h and lysed using the radio immunoprecipitation assay (RIPA) Protein Extraction Kit (Beyotime, China). Protein concentration was quantified using an enhanced BCA Protein Assay Kit (Beyotime, China). Proteins were denatured by incubation in a 100 °C water bath for 5 min, separated using SDS-PAGE, and transferred onto a PVDF membrane (Millipore, USA). The membrane was blocked with 5% BSA and incubated with the following primary antibodies at 4 °C for 12 h: CDK2 (Proteintech, 1:2,000, cat. no. 10122-1-AP), CDK4 (Proteintech, 1:1,000, cat. no. 11026-1-AP), CDK6 (CST, 1:1,000, cat. no.13331T), cyclin D1 (CST, 1:1,000, cat. no. 2922S), caspase 8 (Proteintech, 1:2000, cat. no. 66093-1-Ig), Bax (ZenBio, 1:3,000, cat. no. R22708), MCL1 (ZenBio, 1:1,000, cat. no. R22875), Bcl-2 (ZenBio, 1:1,000, cat. no. HR60015), E-cadherin (CST, 1:1,000, cat. no. 3195T), N-cadherin (Proteintech, 1:2000, cat. no. 22018-1-A), vimentin (ZenBio, 1:1,000, cat. no. R22775), MMP2 (ZenBio,1:1,000, cat. no. R380817), TNFα (Proteintech, 1:1,000, cat. no. 17590-1-AP), P65 (ZenBio, 1:1,000, cat. no. R380172), p-P65 (ZenBio,1:1,000, cat. no. R380172), IKBα (Proteintech, 1:2000, cat. no. 10268-1-AP), p-IKBα (Proteintech, 1:1,000, cat. no. 82349-1-RR), and OGDH (Proteintech, 1:2000, cat. no. 15212-1-AP). Then, the membranes were incubated with HRP-conjugated secondary antibodies (A0216 and A0208, Beyotime; 1:1,000) at 37 °C for 2 h. Finally, protein bands were visualized using the ECL Reagent (Thermo Scientific, USA). Protein expression was normalized to that of β-actin.

### Cell cycle and apoptosis analyses

Cells were treated with PLE at specific concentrations (10, 20, or 40 μM) for 48 h. For cell cycle analysis, cells were harvested, fixed in pre-cooled 75% ethanol overnight at 4 °C, and stained with 5 μL propidium iodide (PI) for 30 min at 37 °C. DNA content was analyzed by flow cytometry using a FACS (Beckman Coulter, USA), and the distribution of cells in the G0/G1, S, and G2/M phases was determined.

For apoptosis analysis, cells were treated with PLE at specific concentrations (10, 20, or 40 μM), harvested, stained with Annexin V-FITC (BD, USA) and PI according to the manufacturer’s instructions, and incubated at 37 °C for 30 min. Early apoptotic, late apoptotic, and necrotic/dead cell populations were analyzed by flow cytometry using a FACS system (Beckman Coulter, USA).

### TUNEL assay

Cells were treated with PLE (10, 20, or 40 μM) for 48 h, fixed with 4% PFA, and permeabilized with 0.1% Triton X-100. Cell apoptosis was measured using a TUNEL reagent (Dalian Bergolin Biotechnology Co., Ltd.). Next, 50 μL DAPI was added into each well, and the cells were incubated at room temperature for 20 min in the dark. Then, they were washed, imaged, and analyzed under a microscope (Olympus, Japan).

### EdU assay

Proliferation of HCCLM3 and Hep3B cells was assessed using the EdU detection kit (RiboBio, China) after PLE treatment at different concentrations (10, 20, or 40 μM) for 48 h. Next, the cells were treated with EdU-labeling medium (50 μM) for 2 h, fixed in 4% PFA for 30 min, and treated with glycine (2 mg/mL) for neutralization. Finally, the cells were stained with the Apollo fluorescent dye for 1 h, permeabilized with 0.1% Triton X-100, and Hoechst 33342 was added to stain the nucleus. Then, the cells were washed, imaged, and counted.

### Spheroid formation assay

The cells were seeded at a concentration of 1 × 104 cells per well in DMEM/F-12 (Gibco, USA) supplemented with B-27 (Gibco, USA), 1% N-2 (Gibco, USA), bFGF (10 ng/mL, Gibco, USA), and EGF (20 ng/mL, Gibco, USA) for 24 h. Then, the cells were treated with PLE (10, 20, or 40 μM) for 48 h, DMEM/F12 was replaced with fresh medium, and the resulting spheres were imaged and counted after 15 days of culture.

### Analysis of cell bioenergetics

The oxygen consumption rate (OCR) was measured using a Seahorse XFe96 Analyzer (Agilent, USA). HCCLM3 and Hep3B cells were treated with PLE (40 μM) for 48 h, seeded into Seahorse XF96 cell culture microplates at a density of 5 × 10^4^ cells per well, and incubated for 12 h to allow attachment. Then, the cells were washed. The culture medium was replaced with 0.5 mL XFassay medium and incubated in the absence of CO_2_ for 1 h. Plates were transferred to the Seahorse analyzer for measurement. During the assay, 1.5 μM oligomycin, 1.5 μM FCCP, and 0.5 μM rotenone/antimycin A were sequentially injected to the cell culture plate according to the manufacturer’s instructions. OCR data were collected and analyzed using Agilent Seahorse Wave software.

### RNA sequencing and qRT-PCR

HCC cells were seeded onto a 6-well plate at a concentration of 1× 10^4^ per well and treated with PLE (40 μM) for 48 h. Total RNA was extracted using TRIzol ® reagent (Tiangen, China). RNA samples (n = 3) were subjected to transcriptomic sequencing and analysis by BGI Co., Ltd. (Shenzhen, China). Differentially expressed genes were identified based on a threshold of |log_2_ fold change| ≥ 1 and −log_10_(P) ≥ 10 and were used for subsequent functional analysis. For qRT-PCR analysis, cDNA was synthesized using the PrimeScript™ RT reagent Kit (TaKaRa, Japan). PCR amplification was performed using SYBR Premix Ex Taq™ II (Thermo, USA). Relative gene expression was calculated using the 2^−ΔΔCT^ method, with β-actin used as the internal control. Primer sequences are listed in Table 1.

**TABLE 1:** Primer sequences for real-time RT-PCR.

Gene	Primer sequences (forward/Reverse)
BCL2	5′-GGTGGGGTCATGTGTGTGG-3′ 5′-CGGTTCAGGTACTCAGTCATCC-3′
BAX	5′-CCTTTTCTACTTTGCCAGCAAAC-3′ 5′-GAGGCCGTCCCAACCAC-3′
Caspase 8	5′-GGATGATGACATGAACCTGCTGGA-3′ 5′-TTGTTGATTTGGGCACAGACTCTT-3′
MCL1	5′-GTAATAACACCAGTACGGACGG-3′ 5′-CCACAAACCCATCCTTGGAAG-3′
CDK2	5′-CCAGGAGTTACTTCTATGCCTGA -3′ 5′-TTCATCCAGGGGAGGTACAAC-3′
CDK4	5′-TCAGCACAGTTCGTGAGGTG-3′ 5′-GTCCATCAGCCGGACAACAT-3′
CDK6	5′-TCTTCATTCACACCGAGTAGTGC -3′ 5′-TGAGGTTAGAGCCATCTGGAAA -3′
Cyclin D1	5′-GCTGCGAAGTGGAAACCATC-3′ 5′-CCTCCTTCTGCACACATTTGAA-3′
E-cadherin	5′-CGAGAGCTACACGTTCACGG-3′ 5′-GGGTGTCGAGGGAAAAATAGG-3′
N-cadherin	5′-AGCCAACCTTAACTGAGGAGT-3′ 5′-GGCAAGTTGATTGGAGGGATG-3′
Vimentin	5′-AGTCCACTGAGTACCGGAGAC-3′ 5′-CATTTCACGCATCTGGCGTTC-3′
MMP2	5′-TACAGGATCATTGGCTACACACC -3′ 5′-GGTCACATCGCTCCAGACT-3′
ACO2	5′-TTGAGCCCAACGAGTACATCC-3′ 5′-GTCCATACACAATCTTCTCCGAG-3′
OGDH	5′-GGCTTCCCAGACTGTTAAGAC-3′ 5′-GCAGAATAGCACCGAATCTGTTG-3′
SUCLG1	5′-GAGCAACGGCTTCTGTCATTT-3′ 5′-TGCTTGACTCGTACCATGTCC-3′
β-actin	5′-GGGAAATCGTGCGTGACATT-3′ 5′-TGCCCAGGAAGGAAGGCT-3′

### RNA interference experiments

Small interfering RNAs (siRNAs) targeting OGDH and negative control siRNAs were designed and synthesized by Tsingke Biotechnology (Beijing, China). Cells were transfected with siRNA using Lipofectamine 3000 (Thermo Fisher, USA) according to the manufacturer’s instructions. Knockdown efficiency was verified at protein levels, and subsequent functional assays were performed. The siRNA oligo sequences are as follows: si-RNA1: sense 5′-UGA​CAA​GUC​UAG​UGA​GAA​UTT-3′, antisense 5′- AUU​CUC​ACU​AGA​CUU​GUC​ATT-3′. si-RNA2: sense 5′-CCA​UUC​CGG​AAG​CCG​UUA​AUU​TT-3′, antisense 3′-AAU​UAA​CGG​CUU​CCG​GAA​UGG​TT-5’. si-Control: sense 5′- UUC​UCC​GAA​CGU​GUC​ACG​UTT-3′, antisense 3′- ACG​UGA​CAC​GUU​CGG​AGA​ATT-5’.

### Plasmid construction and transfection

Cells were transfected with an OGDH overexpression vector (oe-OGDH) using Lipofectamine 3000 (Thermo Fisher, USA) and incubated for 24 h. The transfected cells were lysed, and OGDH protein expression was assessed using Western blotting to confirm overexpression before downstream functional analysis.

### Cell migration assay

Cell migration was assessed using the Transwell assay. HCCLM3 and Hep3B cells were treated with PLE at different concentrations (10, 20, or 40 μM) for 24 h. For the Transwell assay, cells were trypsinized and seeded into the upper chamber of each Transwell insert (1 ×10^4^ cells per well) in 0.2 mL DMEM containing 2% FBS (Millipore, Billerica, MA, USA). The lower chamber was filled with DMEM (0.8 mL) supplemented with 12% FBS as a chemoattractant and incubated for 12 h. Next, the insert was removed, and the cells were fixed with 4% PFA. Non-migrated cells were scraped off, stained with crystal violet, washed, imaged, and quantified.

### Metabolite extraction and LC–MS analysis

Hep3B cells were treated with PLE (40 μM; final DMSO concentration, 0.08%) for 48 h. The cells were then washed twice with pre-chilled PBS on ice and lysed using an ice-cold extraction solvent (1 mL/well; water containing 0.5% formic acid:methanol:acetonitrile at a ratio of 20:40:40). The plate was maintained on ice for 5 min, followed by the addition of 15% NH_4_HCO_3_ (50 µL) to each well. The cells were subsequently scraped, collected, and centrifuged. The supernatant containing metabolites was transferred to a sterile tube and stored at −80 °C until analysis. All samples (n = 6) were analyzed in a randomized sequence to minimize batch effects. Liquid chromatography–mass spectrometry (LC–MS) analysis was performed following the previously described method (Li S. et al., 2022). In brief, the conditions were optimized using an HPLC–ESI–MS system consisting of a Vanquish Horizon UHPLC coupled to a Thermo Q Exactive Plus MS. Chromatographic separation was achieved on an HSS T3 C18 column maintained at 50 °C. The mobile phases were (A) water containing 0.1% formic acid and (B) acetonitrile containing 0.1% formic acid. The flow rate was set at 0.4 mL/min, and the injection volume was 10 μL. Quality control (QC) samples were injected every 10 runs to monitor system stability and reproducibility. The MS data were acquired in both the positive and negative ionization modes, with a resolution of 70,000 (at m/z 200), an automatic gain control target of 3 × 10^6^, and a scan range of 72–1,000 m/z. Raw data were converted to mzXML or mzData format and processed using Metabolomic Analysis and Visualization Engine (MAVEN) for feature detection and peak identification. Data visualization and inspection were performed within MAVEN. Furthermore, statistical and pathway analyses (SMPDB database) were performed using MetaboAnalyst software (V5.0).

### Animal studies

Thirty female nude mice (6 weeks old) were obtained from the Animal Experimental Centre of the Chongqing Medical University and maintained under specific pathogen-free (SPF) conditions. All animal experimental procedures were approved by the Affiliated Hospital of Chongqing Three Gorges Medical College (protocol code SYYZ-A-2303-0018). Hep3B cells (1 × 10^6^ cells per mouse) were subcutaneously injected into the right axillary region. Mice were randomly divided into five groups after 12 days: control, PLE intraperitoneal injection (10, 20, and 30 mg/kg), or positive control (intraperitoneal injection of cisplatin; CDDP, 5 mg/kg). Tumor volume was measured using a Vernier caliper before each administration and calculated using the following formula: tumor volume = [π/6] × length × width^2^. The administrations were repeated eight times. Mice were sacrificed at the end of the experiment via cervical dislocation in accordance with standard procedures (Pan et al., 2022). Tumors were collected, fixed in 4% PFA for at least 48 h, and processed for subsequent experiments.

### Histological analysis

For hematoxylin and eosin (H&E) staining, the collected tumors were fixed, embedded in paraffin, and cut into 8-μm-thick sections. The sections were stained using an H&E Kit (Beyotime, China) according to the manufacturer’s instructions.

For the immunohistochemistry (IHC) assay, paraffin-embedded tumor sections were deparaffinized and rehydrated. Antigen retrieval was performed using a sodium citrate buffer, followed by blocking of non-specific binding sites. The sections were incubated with the anti-Ki67 primary antibody (Proteintech, China) at 4 °C overnight. The sections were washed three times in PBS, incubated with a secondary antibody at 37 °C for 30 min, stained with 3,3′-diaminobenzidine, and imaged under a microscope.

### Statistical analysis

Statistical analysis was performed using GraphPad Prism 8.0 (USA). One‐way ANOVA was used to compare multiple groups followed by Dunnett’s multiple comparisons test, whereas Student’s *t*-tests were used to compare two groups. The results are presented as mean ± standard deviation (SD) of at least three independent biological replicates, each including technical replicates. A value of *P* < 0.05 was considered statistically significant, whereas *P* < 0.01 was considered extremely statistically significant.

## Results

### PLE inhibits HCC cell growth and viability *in vitro*

**FIGURE 1:**
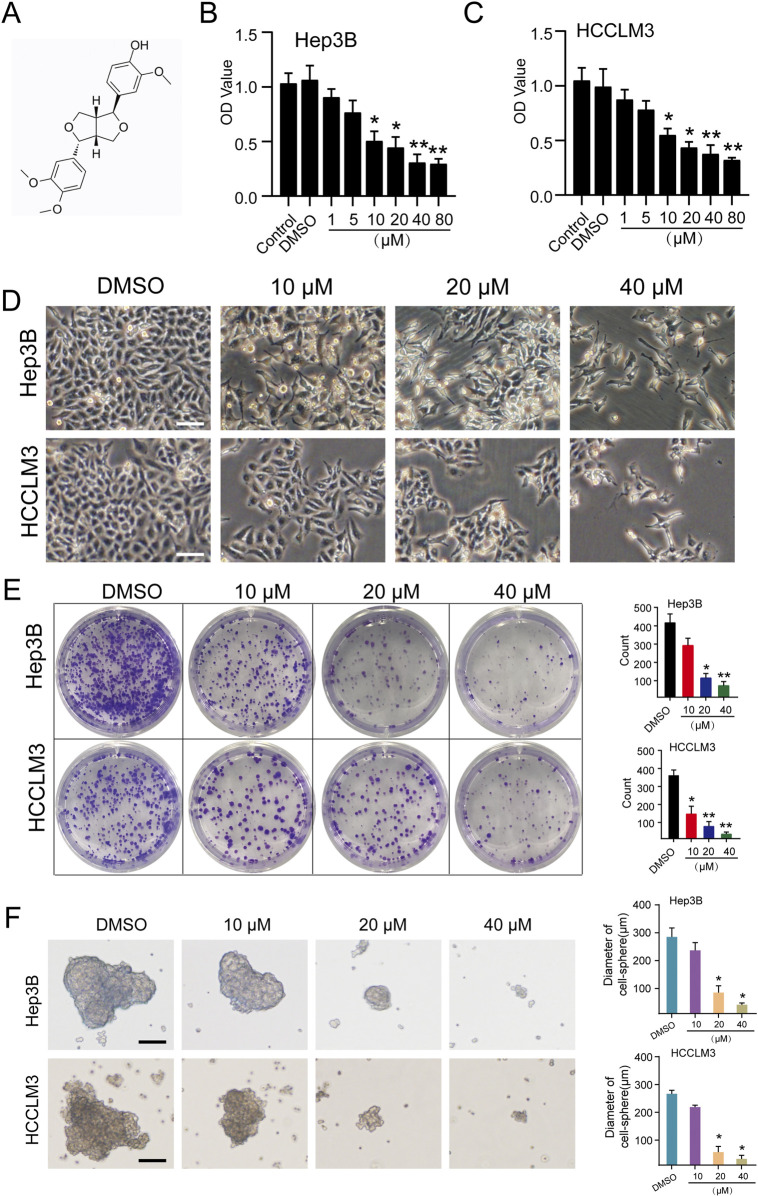
PLE inhibits the viability and proliferation of hepatocellular carcinoma (HCC) cells. **(A)** Molecular structure of PLE. **(B,C)** Viability of PLE-treated Hep3B and HCCLM3 cells analyzed using the CCK8 assay. n = 6 **(D)** Morphology of HCC cells treated with PLE for 48 h. Scale bar = 25 µm. **(E)** Colony formation of HCC cells treated with PLE. n = 6 **(F)** Sphere formation abilities of HCC cells with PLE for 48 h (scale bar = 200 μm). n = 5. One‐way analysis of variance (ANOVA) was employed for multiple comparisons, whereas *t* tests were used for two‐group comparisons. *p < 0.05 and **p < 0.01 indicate significant differences compared with the control group.

The chemical structure of PLE is shown in Figure 1A. PLE treatment reduced the viability of HCC cells (Figures 1B,C). Morphological alterations were observed, with an increased number of exfoliated cells detected in a dose-dependent manner (Figure 1D). Furthermore, PLE treatment reduced colony formation in HCC cells, indicating decreased clonogenic capacity (Figure 1E). In addition, the volume of HCC cell spheres was significantly decreased (Figure 1F). These results indicated that PLE significantly suppressed HCC cell growth and viability.

### PLE regulates gene expression and pathway enrichment in HCCLM3 cells

**FIGURE 2:**
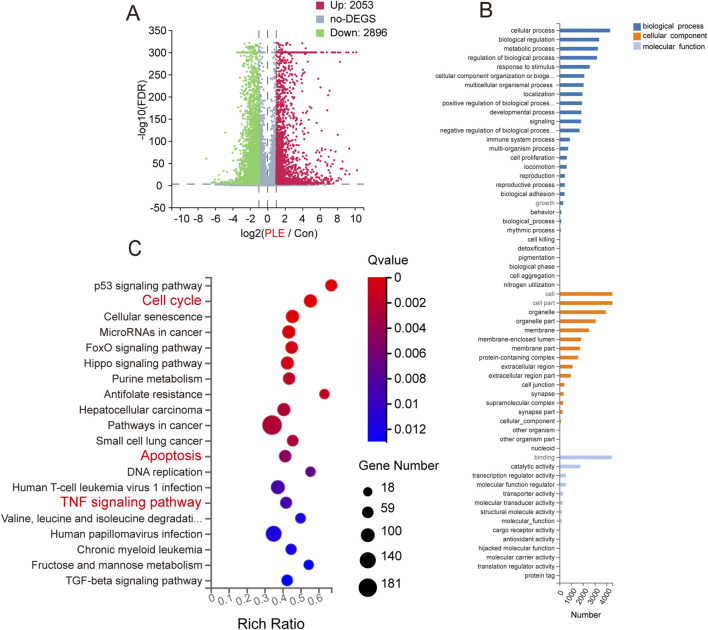
Gene expression profiles following PLE treatment. **(A)** Significantly differentially expressed genes (DEGs) are shown in the volcano plot, with 2,053 upregulated and 2,896 downregulated genes. **(B)** Gene Ontology annotation analysis of PLE (40 μM)-treated HCCLM3 cells compared with control cells. **(C)** Kyoto Encyclopedia of Genes and Genomes (KEGG) pathway enrichment analysis. The larger the P-value (−log10), the higher the enrichment. Important pathways involved in metabolic pathways after treatment of cells with PLE. n = 3.

RNA-seq revealed PLE-mediated gene expression in HCCLM3 cells. PLE treatment resulted in the upregulation of 2,053 genes and downregulation of 2,896 genes (Figure 2A). The Gene Ontology (GO) analysis showed enrichment in biological regulation and metabolic processes (Figure 2B). Kyoto Encyclopedia of Genes and Genomes (KEGG) pathway analysis further indicated significant enrichment of cell cycle, apoptosis, and TNF signaling pathways (Figure 2C). These results indicated that PLE modulated gene expression and affected key regulatory pathways in HCC cells.

### PLE inhibits cell proliferation of Hep3B and HCCLM3 cells *in vitro*

**FIGURE 3:**
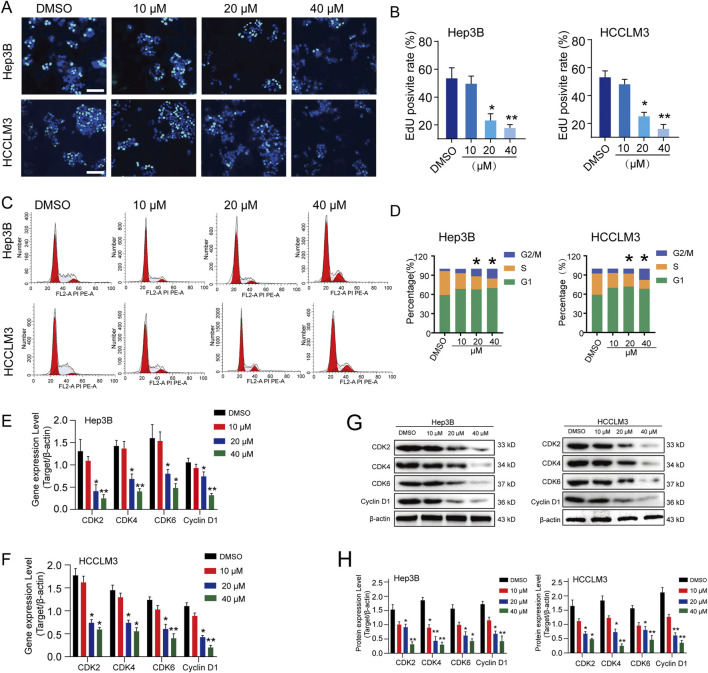
PLE inhibits the proliferation of HCC cells by regulating the cell cycle. **(A,B)** Cell proliferation from the EdU assay of HCC cells following treatment with PLE for 48 h. n = 5. **(C,D)** Cell cycle analysis of HCC cells by flow cytometry following treatment with PLE for 48 h. n = 4. **(E,F)** Gene expression of CDK2, CDK4, CDK6, and cyclin D1 in HCC cells treated with PLE for 48 h. The relative expression levels were analyzed using the 2^−ΔΔct^ method. n = 4. **(G)** Protein expression of CDK2, CDK4, CDK6, and cyclin D1 in HCC cells treated with PLE for 48 h. n = 3. **(H)** Quantitative analysis of the protein bands in **(G)**. One‐way analysis of variance (ANOVA) was employed for multiple comparisons, whereas *t* tests were used for two‐group comparisons. *p < 0.05 and **p < 0.01 indicate significant differences compared with the control group.

PLE treatment markedly decreased the number of proliferating cells (Figures 3A,B). In addition, PLE significantly reduced the proportion of HCC cells entering the S phase. PLE reduced the percentage of Hep3B and HCCLM3 cells in the S phase by approximately 19.18% and 22.24%, respectively (Figures 3C,D). Consistently, the expression of the S phase regulators CDK2, CDK4, CDK6, and cyclin D1 was reduced by PLE at both mRNA and protein levels (Figures 3E–H). These findings suggested that PLE suppressed HCC cell proliferation by inhibiting cell cycle progression.

### PLE promotes apoptosis of Hep3B and HCCLM3 cells

**FIGURE 4:**
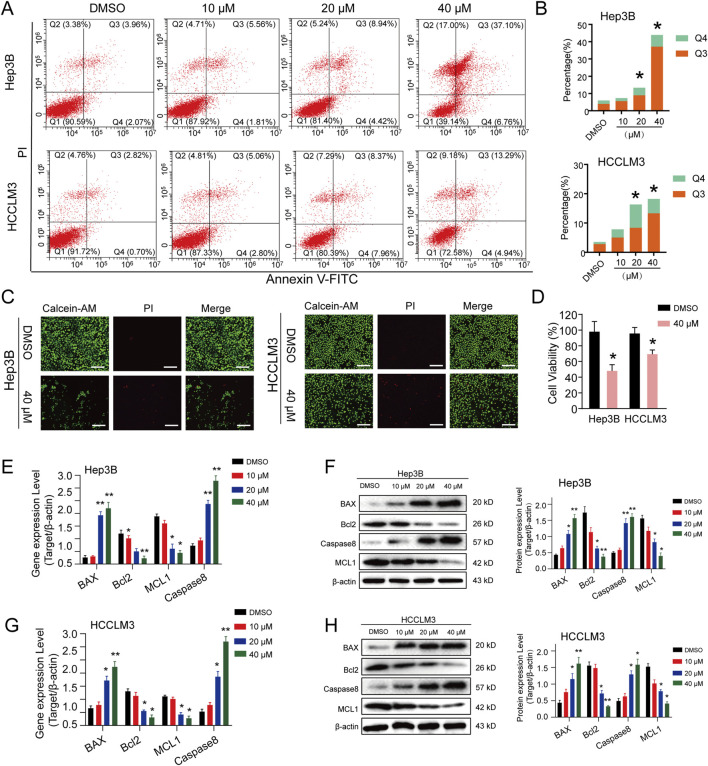
PLE induces apoptosis in HCC cells. **(A,B)** Flow cytometric analysis of apoptosis in HCC cells following treatment with PLE for 48 h. n = 4. **(C,D)** Cell apoptosis from the live/dead staining assay of HCC cells following treatment with PLE for 48 h. n = 5. **(E,G)** Gene expression of MCL1, Bax, caspase 8, and Bcl-2 in HCC cells treated with PLE for 48 h. The relative expression levels were analyzed using the 2^−ΔΔct^ method. n = 4. **(F,H)** Protein expression of MCL1, Bax, caspase 8, and Bcl-2 in HCC cells treated with PLE for 48 h. n = 3. β-actin was used as an internal control. One‐way analysis of variance (ANOVA) was employed for multiple comparisons, whereas *t* tests were used for two‐group comparisons. *p < 0.05 and **p < 0.01 indicate significant differences compared with the control group.

PLE treatment increased HCC cell apoptotic rates in a dose-dependent manner. The apoptotic rates at 40 μM PLE increased by approximately 37.83% and 14.71% in Hep3B and HCCLM3 cells, respectively (Figures 4A,B). Consistently, an increased proportion of dead cells was observed compared with the DMSO-treated group (Figures 4C,D). Furthermore, the expression of apoptosis-related proteins was altered, with a significantly decreased expression of Bcl-2 and MCL1 and increased expression of Bax and caspase 8 at both mRNA and protein levels (Figures 4E–H). These results suggested that PLE promoted the apoptosis of HCC cells *in vitro*.

### PLE suppresses migration of Hep3B and HCCLM3 cells

**FIGURE 5:**
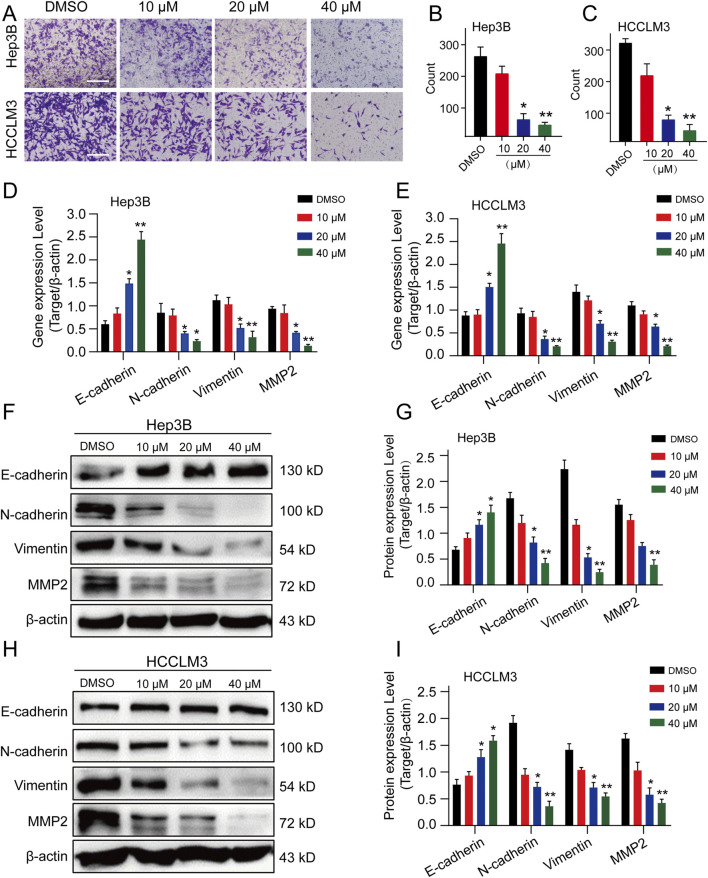
PLE inhibits the migration of HCC cells. **(A–C)** Migration of HCC cells treated with PLE for 24 h. n = 6. **(D,E)** Gene expression of MMP2, E-cadherin, vimentin, and N-cadherin in HCC cells treated with PLE for 24 h. The relative expression levels were analyzed using the 2^−ΔΔct^ method. n = 4. **(F,H)** Protein expression of MMP2, E-cadherin, vimentin, and N-cadherin in HCC cells treated with PLE for 24 h. β-actin was used as an internal control. n = 3. **(G,I)** Quantitative image analysis of the protein bands in **(F,H)**. One‐way analysis of variance (ANOVA) was employed for multiple comparisons, whereas *t* tests were used for two‐group comparisons. *p < 0.05 and **p < 0.01 indicate significant differences compared with the control group.

PLE treatment resulted in a marked reduction in cell migration (Figures 5A–C). Consistently, the expression of migration-related markers was altered, with significantly decreased expression of N-cadherin, vimentin, and MMP2 and increased expression of E-cadherin at both the mRNA and protein levels (Figures 5D–I). These results indicated that PLE inhibited the migratory ability of HCC cells.

### PLE regulates cell proliferation and apoptosis through the TNF signaling pathway in HCC cells

**FIGURE 6:**
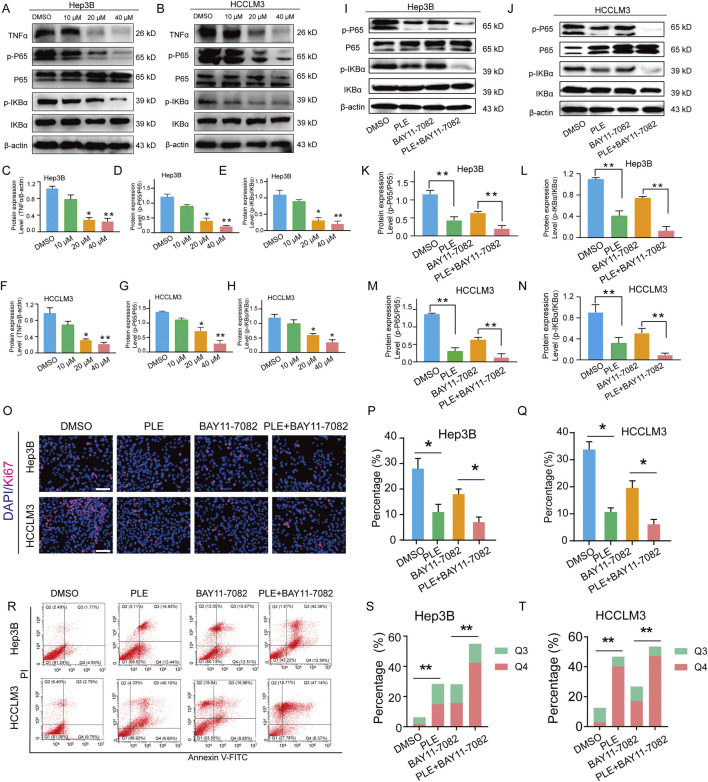
PLE regulates cell proliferation and apoptosis via the TNF pathway in HCC cells. **(A,B)** WB analysis for detecting TNFα, p-P65, and p-IKBα at the protein level in HCC cells. n = 3. **(C,D)** WB analysis for detecting p-P65 and p-IKBα at the protein level after treated with the TNF pathway inhibitor (BAY11-7082) in HCC cells. n = 3. **(E–J)** Quantitative image analysis of the protein bands in **(A,B)**. **(K–N)** Quantitative image analysis of the protein bands in **(C,D)**. **(O–Q)** Ki67 staining assays for cell proliferation after treated with the TNF pathway inhibitor (BAY11-7082, 10 μM) and PLE in HCC cells. Scar bar = 50 μm. n = 5. **(R–T)** Flow cytometric analysis of apoptosis after treated with the TNF pathway inhibitor (BAY11-7082) and PLE in HCC cells. n = 4. One‐way analysis of variance (ANOVA) was employed for multiple comparisons, whereas *t* tests were used for two‐group comparisons. *p < 0.05 and **p < 0.01 indicate significant differences compared with the control group.

PLE treatment reduced the expression of TNF pathway-related proteins, including TNFα, p-P65, and p-IKBα (Figures 6A–H). In addition, co-treatment with the TNF pathway inhibitor BAY11-7082 (10 μM) following PLE treatment further enhanced the effects of PLE in both Hep3B and HCCLM3 cells (Figures 6I–N). Consistently, the inhibition of cell proliferation and the increase in apoptosis by PLE were more pronounced in the presence of BAY11-7082 (Figures 6O–T). These results indicated that the TNF signaling pathway was involved in the regulation of HCC cell proliferation and apoptosis by PLE; however, this is not the only relevant pathway.

### PLE mediates TCA cycle metabolism in HCCLM3 cells

**FIGURE 7:**
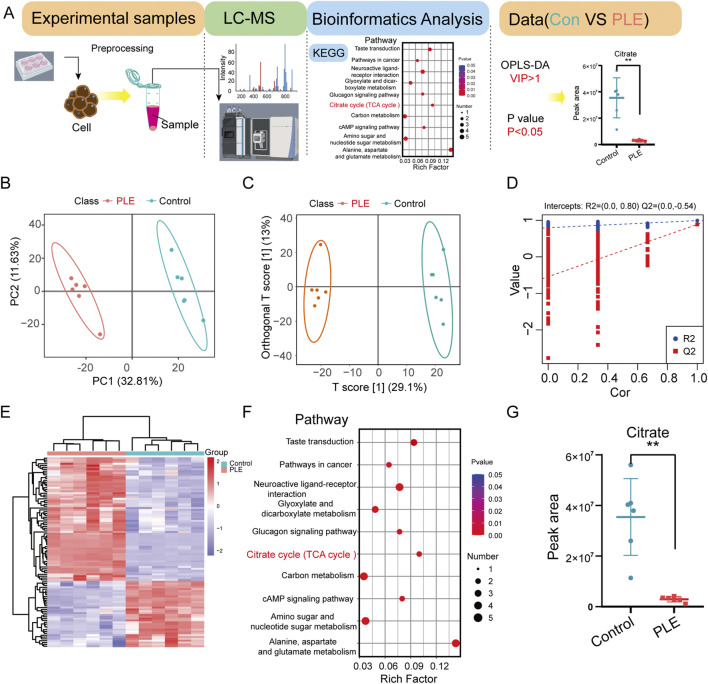
PLE mediates its anti-tumor effects by regulating the TCA cycle metabolic pathway. **(A)** Schematic diagram of the metabolic process based on LC–MS. **(B)** PCA score plot of the control and PLE groups, showing the tight clustering between the two groups. n = 6. **(C)** OPLS-DA score plot of the control and PLE (40 μM) groups, showing the clustering of samples in the training set. **(D)** OPLS-DA verification model of the control and PLE (40 μM) groups. **(E)** Heat map of metabolites between control and PLE (40 μM) groups. Metabolites that increased are shown in red, whereas those that decreased are shown in purple. **(F)** Plots depict the computed metabolic pathways as a function of −log (p) (y-axis) and the key metabolites (x-axis) for key differential metabolites between the control and PLE (40 μM) groups. The size of a circle is proportional to the pathway impact value. **(G)** Metabolites associated with the TCA cycle metabolism altered by PLE (40 μM) treatment in HCCLM3 cells. One‐way analysis of variance (ANOVA) was employed for multiple comparisons, whereas *t* tests were used for two‐group comparisons. *p < 0.05 and **p < 0.01 indicate significant differences compared with the control group.

A schematic representation of the metabolic process is shown in Figure 7A. PLE treatment resulted in distinct metabolic profiles compared with the DMSO-treated group, as demonstrated by principal component analysis (PCA) (Figure 7B). Orthogonal partial least-squares discriminant analysis (OPLS-DA) further revealed differences between the DMSO-treated and PLE-treated groups (Figure 7C), and OPLS-DA model validation suggested an acceptable fit (Figure 7D). Metabolomic profiling revealed that PLE markedly altered the levels of multiple primary metabolites (Figure 7E). Pathway enrichment analysis revealed that the TCA cycle and carbon metabolism pathways were affected (Figure 7F). In particular, citrate levels were significantly reduced following PLE treatment (Figure 7G). These results suggested that PLE modulated TCA cycle metabolism in HCC cells.

### PLE regulates the cell cycle and apoptosis through OGDH in Hep3B and HCCLM3 cells

**FIGURE 8:**
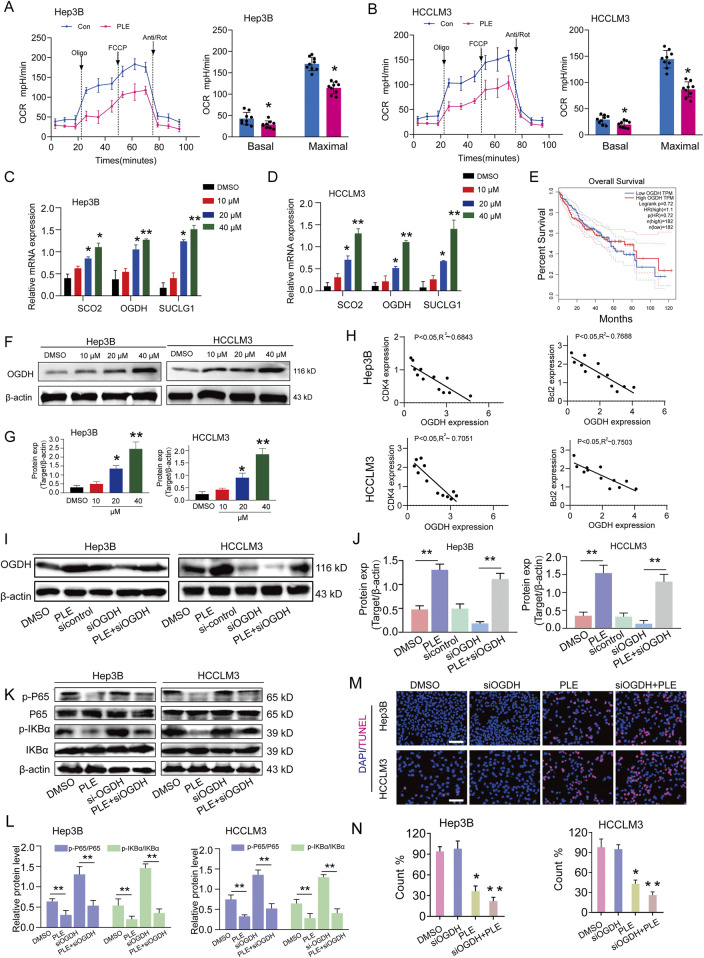
PLE upregulates OGDH expression in HCC cells. **(A,B)** Oxygen consumption rates (OCRs) of HCC cells were measured with or without PLE. n = 6. **(C,D)** Gene expression of SCO2, OGDH, and SUCLG1 in HCC cells treated with PLE for 48 h. The relative expression levels were analyzed using the 2^−ΔΔct^ method. n = 4. **(E)** Kaplan–Meier (K–M) survival analysis of changes in OGDH expression, and prediction of survival and prognosis of patients with HCC. **(F)** OGDH protein expression in HCC cells treated with PLE for 48 h. n = 3. **(G)** Quantitative image analysis of the protein bands in **(F)**. **(H)** Correlation analysis showed that the expression of CDK4 and Bcl-2 highly correlated with that of OGDH. **(I,J)** Western blotting analysis for detecting OGDH at the protein level with PLE or siOGDH for 48 h. n = 3. **(K,L)** Western blotting analysis for detecting p-P65/p-IKBα at the protein level with PLE or siOGDH for 48 h. n = 3. **(M,N)** TUNEL staining assays for cell apoptosis after treated with siOGDH and PLE for 48 h (scale bar = 50 μm). n = 6. One‐way analysis of variance (ANOVA) was employed for multiple comparisons, whereas *t* tests were used for two‐group comparisons. *p < 0.05 and **p < 0.01 indicate significant differences compared with the control group.

PLE treatment significantly inhibited the oxidative phosphorylation in HCC cells (Figures 8A,B). Furthermore, the expression of the TCA cycle-related genes ACO2, SUCLG1, and OGDH was increased (Figures 8C,D). Elevated OGDH expression was associated with improved overall survival in patients with HCC (Figure 8E). Consistently, OGDH protein expression was increased following PLE treatment (Figures 8F,G). Correlation analysis revealed that OGDH expression was negatively associated with the cancer malignancy markers CDK4 and Bcl-2 (Figure 8H). OGDH knockdown significantly reduced its expression, with siRNA2 showing a stronger inhibitory effect than siRNA1 (Supplementary Figures S1A,B). In the subsequent experiments, we used siRNA2 with a better inhibitory effect and named it siOGDH for the mechanism exploration experiments. Functionally, OGDH inhibition significantly reversed the enhancing effect of PLE on OGDH expression in HCC cells (Figures 8I,J). In addition, OGDH knockdown reduced the suppressive effect of PLE on sphere formation (Supplementary Figures S1C,D). Inhibition of OGDH increased p-P65 and p-IKBα expression, while PLE treatment reduced these effects (Figures 8K,L). OGDH knockdown also reduced PLE-induced apoptosis (Figures 8M,N). Furthermore, CDK4 and Bcl-2 expression increased after OGDH inhibition, whereas PLE partially reversed these changes (Supplementary Figures S1E,F). Consistently, OGDH overexpression suppressed p-P65 and p-IKBα expression and inhibited proliferation in HCC cells, and this inhibitory effect was further enhanced by PLE (Supplementary Figure S2). These findings indicated that PLE regulated cell proliferation and apoptosis in HCC cells, at least in part, through the modulation of OGDH.

### PLE inhibits tumor growth *in vivo*

**FIGURE 9:**
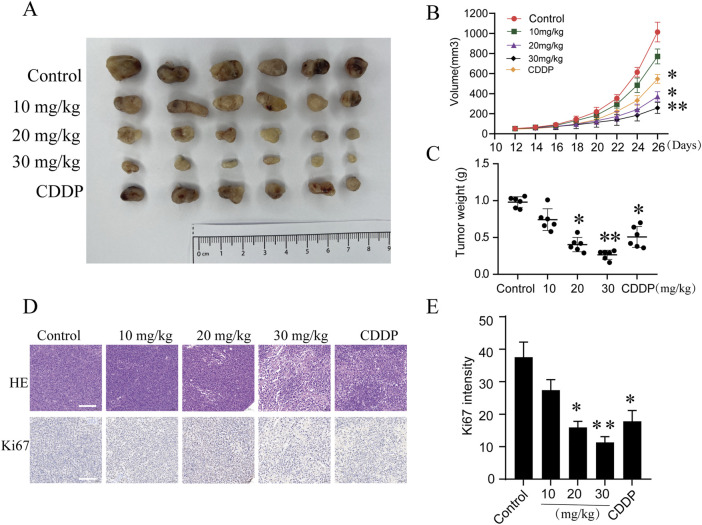
PLE inhibits tumor growth *in vivo*. Thirty nude mice were randomly divided into five groups, namely, control, PLE (10, 20, and 30 mg/kg), and positive control (CDDP, 5 mg/kg) groups. The mice were injected once in 2 days. n = 6. **(A)** Images of tumors in the indicated mice. **(B)** Tumor volume in the indicated mice. **(C)** Tumor weight in the indicated mice. **(D)** Hematoxylin and eosin and immunohistochemical (IHC) staining of Ki67 in the indicated tumors. **(E)** Quantitative image analysis of Ki67 expression in tumors shown in **(D)**. Scale bar = 100 μm. One‐way analysis of variance (ANOVA) was employed for multiple comparisons, whereas *t* tests were used for two‐group comparisons. *p < 0.05 and **p < 0.01 indicate significant differences compared with the control group.

Tumor weight and volume after seven PLE treatments were significantly reduced in both PLE and CDDP groups compared with those in the control group (Figures 9A–C). Histological analysis further showed reduced tumor cell density and decreased Ki67 expression in the PLE-treated group (30 mg/kg) (Figures 9D,E). These results suggested that PLE attenuated tumor growth *in vivo*, consistent with the *in vitro* findings.

## Discussion

HCC remains the main cause of cancer-related mortality worldwide, with limited therapeutic options and poor long-term survival (Norero and Dufour, 2023). Although chemotherapeutic drugs are currently used in clinical practice, their efficacy is often constrained by toxicity and the development of resistance (Lyu et al., 2018). Hence, the development of novel therapeutic agents for HCC is a critical unmet medical need. Phytochemicals are widely used for the treatment of cancers, cardiovascular disease, and obesity because of their low systemic toxicity and high efficiency (Al-Ishaq et al., 2020). The advancement of studies on the antitumor functions of natural plant products revealed that several active natural plant products provide promising effects in the treatment of cancer (Feng et al., 2022; Li L. et al., 2022). In addition, several small natural plant compounds are widely used in HCC treatment. Eupalinolide A exhibits anti-cancer effects by inducing autophagy, controlled by reactive oxygen species (ROS) and ERK signaling activation in liver cancer cells (Zhang et al., 2022). Anemoside B4 inhibits proliferation and induces apoptosis and autophagy in SMMC-7721 cells (Xue et al., 2019). PLE, an active ingredient obtained from *F. fructus*, has shown hepatoprotective effects in recent years (Song et al., 2018). PLE decreases liver fibrosis by suppressing LPS-induced pro-inflammatory response and activating LX2 cells through the TLR4/MyD88/NF-κB signaling pathway (Wang et al., 2021). In this context, we demonstrated the antitumor effects of PLE in HCC models. Specifically, PLE reduced the viability, proliferation, and migration of HCC cells, promoted apoptosis *in vitro*, and inhibited tumor growth *in vivo*. PLE concentrations used in this study fall within the range reported for bioactive phytochemicals in preclinical models. Although direct pharmacokinetic data for PLE are currently unavailable, studies on structurally related natural compounds suggest that low micromolar plasma concentrations may be achievable (Kongratanapasert et al., 2024; Grimm et al., 2006). Nevertheless, dedicated pharmacokinetic studies are required to determine the clinical relevance of these findings.

TNF-α is as a crucial regulator of inflammatory signaling and is involved in tumor progression through the modulation of proliferation, apoptosis, and the tumor microenvironment (Cruceriu et al., 2020). Dysregulation of the TNF-α/NF-kB signaling axis is frequently observed in cancer and contributes to malignant phenotypes (Meng et al., 2021). TNF-α can inhibit cell metastasis in gastric cancer by affecting the epithelial–mesenchymal transition process of cells (Zhou et al., 2020). Our transcriptomic results indicated that PLE mediated gene expression by regulating the TNF pathway. Further experimental results suggested that PLE decreased TNF-α expression in HCC cells. Several pieces of evidence show that NF-κB acts as a key mediator of inflammatory transduction cascade reaction, and the activity of NF-κB is regulated by TNF-α and IL-1 (Kim et al., 2018). Excessive activation of the NF-κB pathway is frequently detected in a variety of tumors (Mirzaei et al., 2022). The TNF signaling pathway is inherently pleiotropic—TNFα can induce both pro-inflammatory signals and pro-apoptotic signals, depending on cellular context and the formation of distinct signaling complexes (Gough and Myles, 2020). Studies have shown that in HCC, the NF-κB pathway downstream of TNF-α can promote cell survival (Manohar, 2024; Saitou et al., 2005). Inhibition of the TNF signaling pathway would be expected to relieve anti-apoptotic signals and sensitize cells to apoptosis, further promoting cell apoptosis. In addition, research has reported that Compound 17 induced apoptosis by inhibiting the TNF signaling pathway and modulating apoptosis-related proteins in lung Cancer (Zhang et al., 2026). These findings provide direct evidence for our observation that PLE promotes apoptotic susceptibility by inhibiting the TNF signaling pathway. In the present study, PLE was associated with reduced activation of TNF pathway components, including decreased expression of TNF-α and reduced phosphorylation of P65 and IkBa. These findings, together with the enhanced effects observed upon TNF signaling pathway inhibition, supported the involvement of this pathway in mediating the biological effects of PLE. These results were consistent with previous reports indicating that PLE modulates inflammatory signaling pathways, including NF-κB, and extended these observations to HCC by linking TNF pathway regulation to antiproliferative and pro-apoptotic effects.

Metabolic reprogramming is a hallmark of cancer, enabling tumor cells to sustain growth and adapt to environmental stress (Martínez-Reyes and Chandel, 2021). Although glycolysis has historically been emphasized, recent studies highlight the importance of the TCA cycle in supporting tumor bioenergetics and biosynthesis (Cummins et al., 2014; Anderson et al., 2018). We demonstrated that PLE significantly altered metabolic profiles in HCC cells, with enrichment of TCA-cycle-related pathways and significant changes in key metabolites, including reduced citrate levels. Citrate is also involved in cellular metabolism, supporting tumor growth and proliferation (Petillo et al., 2020). Intracellular citrate levels can mediate the behavior of tumors and immune cells by enhancing cancer cell apoptosis. This improves immune cell response, further enhancing cancer immunotherapy (Huang et al., 2020). The results of this study indicated that PLE interfered with metabolic pathways that are critical for tumor maintenance, thereby contributing to its antitumor effects. However, the broader impact of these metabolic alterations on tumor progression and the tumor microenvironment requires further investigation. The OGDH complex (OGDHC) is a rate-limiting enzyme in the TCA cycle, which regulates metabolic flux in cancer cells based on resource and energy requirements (Pardee et al., 2014; Hosios et al., 2016). OGDH, the E1 subunit of OGDHC, participates in metabolic reprogramming of tumor cells (Vatrinet et al., 2017). OGDH has been implicated in the modulation of cancer cell fate across multiple cancer types, including cell viability and migration (Wang et al., 2025; Ostrow et al., 2009). In this study, PLE was associated with increased OGDH expression, which correlated with reduced expression of proliferation- and survival-related markers. Functional experiments further indicated that the modulation of OGDH levels influenced the effects of PLE on cell cycle progression and apoptosis, supporting a mechanistic link between metabolic regulation and cellular outcomes. These findings identified OGDH as a key mediator of the observed effects and suggested that targeting metabolic enzymes may represent an effective strategy for modulating tumor behavior. Here, we demonstrated that PLE regulates the TNF signaling pathway through OGDH in HCC cells. To our knowledge, this is the first evidence establishing a functional axis between PLE, OGDH, and TNF-driven inflammation in HCC. However, the downstream molecular mechanisms remain to be elucidated.

Collectively, in the present study, we demonstrated that PLE exerts an antitumor effect in HCC through the coordinated regulation of the TNF signaling pathway and TCA cycle metabolism. The identification of OGDH as a potential mediator of these effects highlights a link between metabolic reprogramming and signaling pathway modulation. Although these findings support the potential of PLE as a promising antitumor drug candidate, further studies are required to validate its efficacy in preclinical models and to characterize its pharmacokinetic and safety profile prior to clinical translation.

## Data Availability

The datasets presented in this study can be found in online repositories. The names of the repository/repositories and accession number(s) can be found below: https://www.ncbi.nlm.nih.gov/, PRJNA1002636.
