# Supplementary material for: Phillygenin suppresses hepatocellular carcinoma progression by modulating the TNF signaling pathway and TCA cycle metabolism
Source: Front Pharmacol. 2026 Jun 12;17:1854265. doi: 10.3389/fphar.2026.1854265 (PMC13308294; doi:10.3389/fphar.2026.1854265)
Supplement: Supplementary file 1 [file DataSheet1.docx]

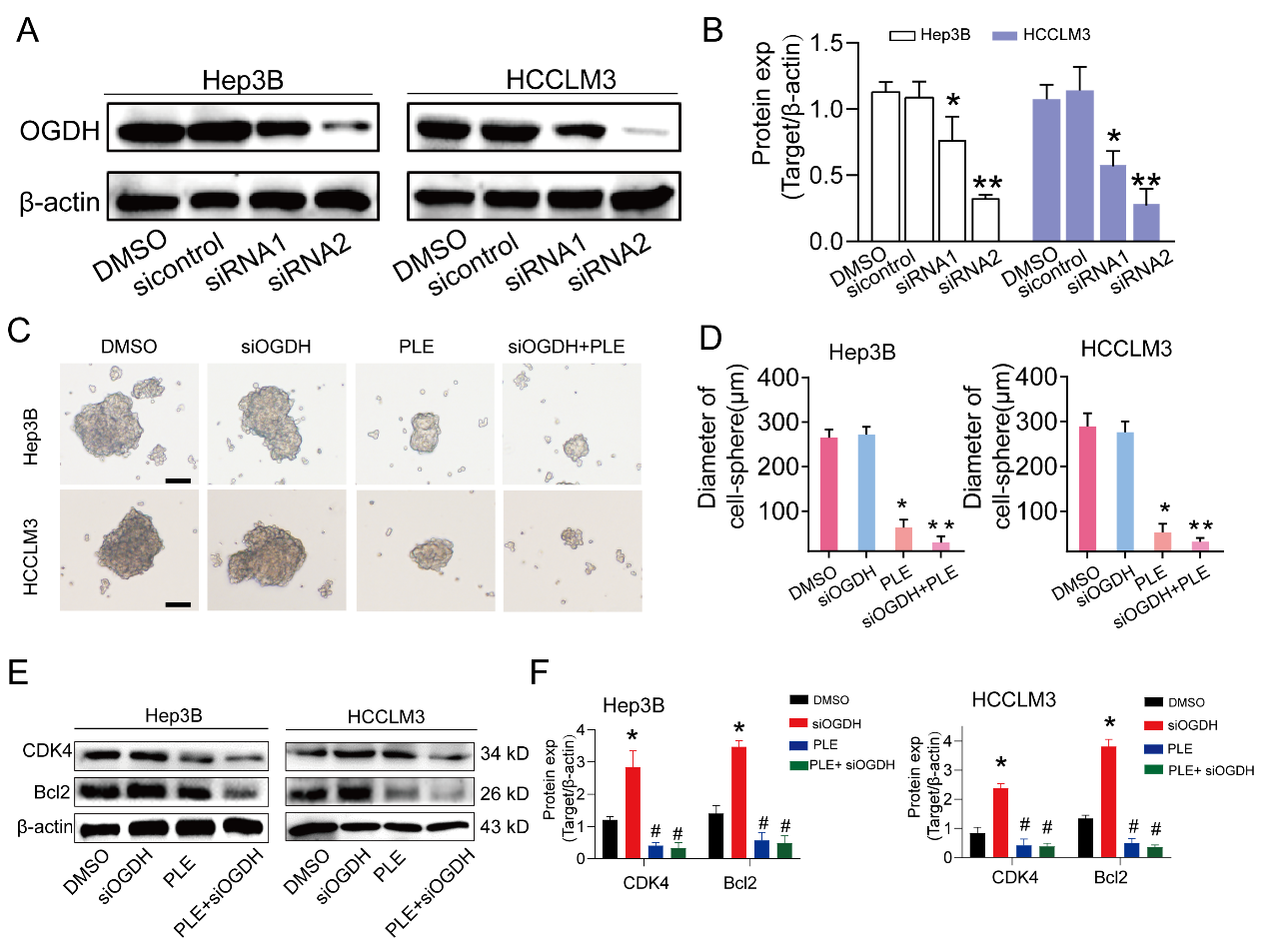


Figure S1 OGDH knockdown suppresses expression of OGDH in HCC cells. (A, B) Western blotting analysis for detecting OGDH at the protein level with two different siRNA sequences for 48 h. n=3. (C, D) Sphere formation abilities of HCC cells with siOGDH and PLE for 48 h (scale bar = 200 μm). n=4. (E, F) TUNEL staining assays for cell apoptosis after treated with siOGDH and PLE for 48 h (scale bar = 100 μm). n=6. One‐way analysis of variance (ANOVA) was employed for multiple comparisons, whereas *t* tests were used for two‐group comparisons. * *p* < 0.05; * * *p* < 0.01(^#^*p* < 0.05, compared to siOGDH group) indicate significant differences compared with the control group.


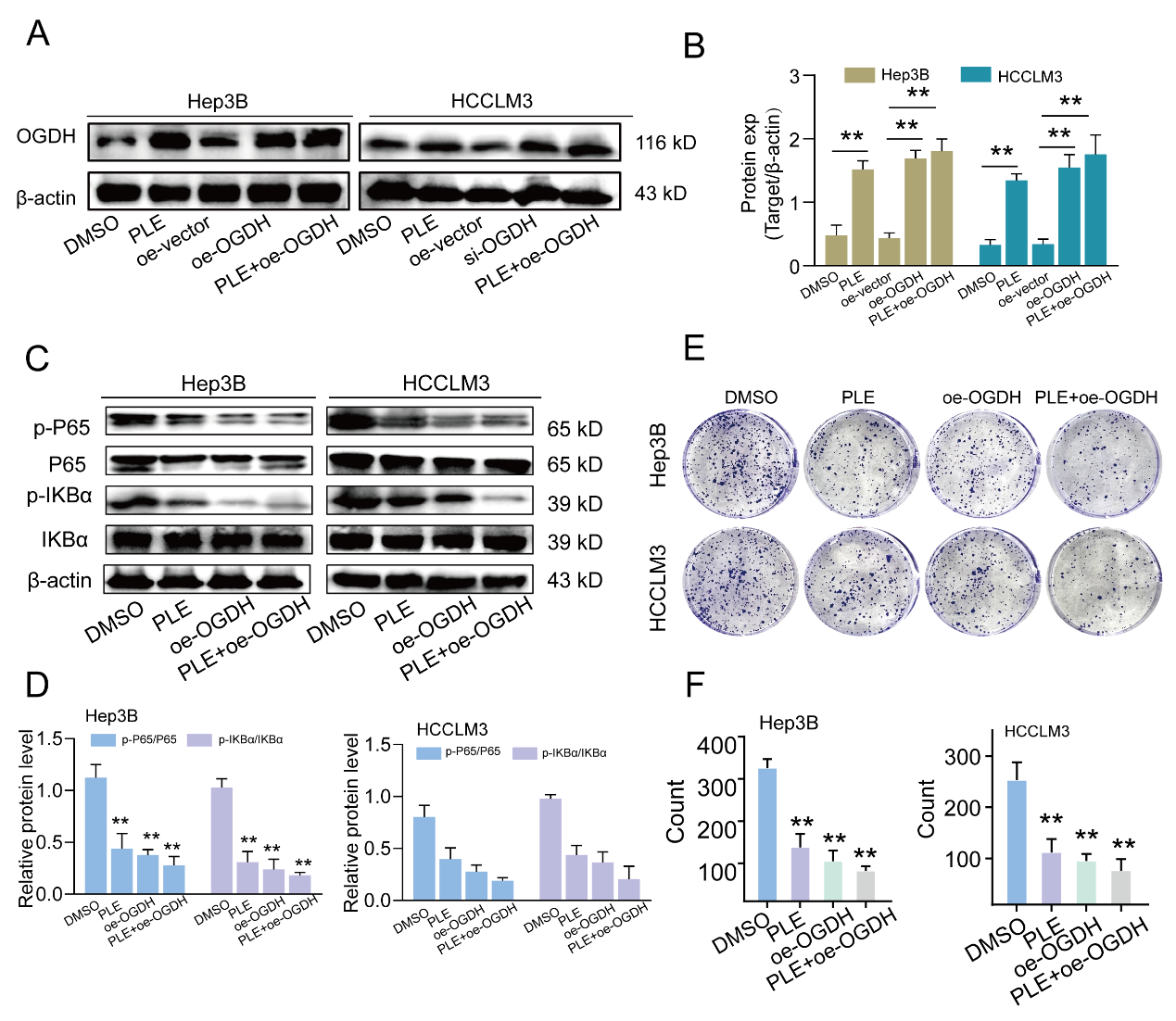


Figure S2 OGDH overexpression promotes expression of OGDH in HCC cells. (A, B) Western blotting analysis for detecting OGDH at the protein level with PLE or oe-OGDH for 48 h. n=3. (C, D) Western blotting analysis for detecting p-P65/ p-IKBα at the protein level with PLE or oe-OGDH. n=3. (E, F) Clone formation of HCC cells after treated with siOGDH and PLE for 48 h. n=4. One‐way analysis of variance (ANOVA) was employed for multiple comparisons, whereas *t* tests were used for two‐group comparisons. * *p* < 0.05; * * *p* < 0.01 indicate significant differences compared with the control group.
